# Bacteria Associated With *Phaeocystis globosa* and Their Influence on Colony Formation

**DOI:** 10.3389/fmicb.2022.826602

**Published:** 2022-02-17

**Authors:** Shuaishuai Xu, Xiaodong Wang, Jie Liu, Fengli Zhou, Kangli Guo, Songze Chen, Zhao-hui Wang, Yan Wang

**Affiliations:** ^1^College of Life Science and Technology, Jinan University, Guangzhou, China; ^2^Department of Ocean Science and Engineering, Southern University of Science and Technology, Shenzhen, China

**Keywords:** *Phaeocystis globosa*, associated bacteria, colony formation, *Marinobacter*, composition

## Abstract

*Phaeocystis globosa* (*P. globosa*) is one of the dominant algae during harmful algal blooms (HABs) in coastal regions of Southern China. *P. globosa* exhibits complex heteromorphic life cycles that could switch between solitary cells and colonies. The ecological success of *P. globosa* has been attributed to its colony formation, although underlying mechanisms remain unknown. Here, we investigated different bacterial communities associated with *P. globosa* colonies and their influence on colony formation of two *P. globosa* strains isolated from coastal waters of Guangxi (GX) and Shantou (ST). Eight operational taxonomic units (OTUs) were observed in ST co-cultures and were identified as biomarkers based on Linear discriminant analysis Effect Size (LEfSe) analysis, while seven biomarkers were identified in *P*. *globosa* GX co-cultures. Bacterial communities associated with the *P. globosa* GX were more diverse than those of the ST strain. The most dominant phylum in the two co-cultures was Proteobacteria, within which *Marinobacter* was the most abundant genus in both GX and ST co-cultures. Bacteroidota were only observed in the GX co-cultures and Planctomycetota were only observed in the ST co-cultures. Co-culture experiments revealed that *P*. *globosa* colony formation was not influenced by low and medium cell densities of *Marinobacter* sp. GS7, but was inhibited by high cell densities of *Marinobacter* sp. GS7. Overall, these results indicated that the associated bacteria are selected by different *P*. *globosa* strains, which may affect the colony formation and development of *P. globosa*.

## Introduction

The *Phaeocystis* are globally distributed marine algae, which cause frequent coastal harmful algal blooms and play important roles in carbon and sulfur biogeochemical cycling (Schoemann et al., 2005; Verity et al., 2007). Owing to their negative effects on marine ecosystems, fisheries, and local economies, the bloom development of *Phaeocystis* has gained much attention in recent decades (Schoemann et al., 2005). *Phaeocystis* species, such as *Phaeocystis globosa*, *Phaeocystis pouchetii*, and *Phaeocystis antarctica* have all been reported to form extensive colony blooms in many regions including in tropic and polar waters (Schoemann et al., 2005).

*Phaeocystis* exhibit complex polymorphic life cycles involving solitary cell and colony stages, wherein solitary cells are generally 3–9 μm and colonies are usually several mm in diameter (Rousseau et al., 1994). Extraordinarily large *Phaeocystis* colonies (up to 3 cm in diameter) have also been found in coastal waters of South China (Qi et al., 2004; Smith et al., 2014).

Colony formation partially underlies the success of *Phaeocystis* in marine ecosystems (Rousseau et al., 1994; Hamm, 2000). Solitary cells are generally consumed by small grazers (Tang et al., 2001), while colonies are ingested by zooplankton to a lesser degree due to their tough exteriors and size mismatches between colonies and grazers (Hamm et al., 1999; Jakobsen and Tang, 2002). Thus, colony formation protects *Phaeocystis* cells from predation and thus significantly decreases mortality (Hamm et al., 1999). Several abiotic and biological factors have been proposed to affect the colony formation of *Phaeocystis*, such as light exposure (Wang et al., 2014), macronutrient levels (Wang et al., 2010), temperatures (Wang et al., 2010), and zooplankton grazing (Jakobsen and Tang, 2002), or combinations of these factors, while the molecular mechanisms underlying colony formation remain enigmatic (Verity et al., 2007).

Colonies largely comprise polysaccharides (Hamm et al., 1999), which provide a carbon source for surrounding bacterial populations (Dutz and Koski, 2006; Verity et al., 2007; Wemheuer et al., 2015). Previous studies have shown dynamic bacterial community compositions during *P. globosa* blooms (Li et al., 2020). For example, in the course of *Phaeocystis* blooms in the southern North Sea, bacterial diversity decreased significantly and Gammaproteobacteria became more abundant (Wemheuer et al., 2014, 2015). During the demise of a *Phaeocystis* spring bloom in the North Sea, Bacteroidota abundances increased sharply, which might be involved in mucopolysaccharide degradation (Alderkamp et al., 2006). Similar trends were also observed in the coastal blooms of South China. For instance, Li et al. (2020) found that bacterial diversity of free-living bacteria was lower during *P*. *globosa* marine blooms, while *Marinobacterium*, *Erythrobacter*, and *Persicobacter* became dominant in the terminal stage of *P*. *globosa* blooms. Zhu et al. (2021) suggested that seawater bacterial richness and diversity were significantly lower in comparison to *P*. *globosa* intracolonial fluids during *P*. *globosa* blooms (Zhu et al., 2021). Despite these observations, the potential effects of bacterioplankton on *P*. *globosa* colony formation remain enigmatic.

Here, bacterial communities associated with *P. globosa* isolated from coastal waters were investigated and their effects on *P. globosa* colony formation were explored *via* co-culture experiments. Specifically, this study aimed to address: (i) how different *P*. *globosa* strains influence bacterioplankton composition and diversity, (ii) the identity of bacterial species associated with *P*. *globosa* colony development, and (iii) the potential bacterial influences on *P. globosa* colony formation.

## Materials and Methods

### Bacterial Growth, Isolation, and Classification

*Phaeocystis globosa* Guangxi (GX) and Shantou (ST) strains were isolated from coastal waters of Guangxi in 2017 and Shantou in 2003, respectively. Bacterial cells were isolated from the exponential stages of *P*. *globosa* GX and ST strain growth by serially diluting 1.0 ml aliquots of cultures into sterile seawater. After serial dilutions, 100 μl of the 10^–2^ to 10^–7^ dilutions were spread onto agar plates of marine agar 2216 (MA; BD Difco) (Yang et al., 2021). Plates were then incubated at 20°C for 7 days in the dark. Bacterial colonies exhibiting different morphological characteristics were isolated and stored in marine broth 2216 (MB, BD Difco) supplemented with 20% glycerol to form stocks that were stored at −80°C for future experiments (Xu et al., 2021a,b).

Bacteria grown on marine agar plates (MA; BD Difco) were incubated at 20°C in the dark with shaking at 200 revolutions per minute (rpm). To identify the isolated bacteria, single colonies were cultured in marine broth after incubating for 72 h in the dark, followed by centrifugation of cells at 10,000 rpm for 1 min. The centrifuged supernatants were removed and DNA was extracted from the pellets using a TIANGEN Bacterial DNA extraction kit according to the manufacturer’s instructions. The 16S rRNA genes from bacterial isolates were then amplified using universal 16S rRNA primers (27F, 1492R) (Frank et al., 2008) and a Green Tap amplification kit (Vazyme, China). Amplicons were sequenced at Sangon Biotech (Shanghai) Co., Ltd. (Shanghai, China). *Marinobacter* sp. GS7 cells were fixed using glutaraldehyde and then photographed using a scanning electron microscope (Zeiss ULTRA™ 55, Carl Zeiss Inc., Oberkochen, Germany).

Sequence alignments were generated for the 16S rRNA genes using the EzBioCloud server^1^ platform. Phylogenetic inference was then conducted using the Maximum-Likelihood method in Mega X and node support was evaluated with 1,000 bootstrap replicates (Kumar et al., 2018).

### *Phaeocystis globosa* Growth

Cultures of *P*. *globosa* GX and ST strains were maintained in the exponential growth stage *via* regular dilution with f/2 medium, and were grown in f/2 medium at 20°C with 12 h light: 12 h dark diurnal cycles (100 μmol m^–2^ s^–1^) (Liang et al., 2020).

### Axenic *Phaeocystis globosa* Culture Generation

To generate axenic cultures, solitary *P. globosa* cells were separated by filtering culture stocks through 10 μm filters. The cells were centrifuged at 2,000 rpm for 5 min, then quickly rinsed with sterile f/2 medium twice and washed for 1 min in sterile media containing 20 μg ml^–1^ Triton X-100 (Amin et al., 2015). Solitary cells were subsequently washed off the filter by gentle shaking into sterile media containing a suite of antibiotics (per milliliter: 5 μg penicillin, 10 μg streptomycin, 0.1 mg kanamycin, and 1 mg ampicillin). Cells were then incubated in antibiotic-containing media for 48 h under equivalent growth conditions. Finally, 20 ml of antibiotic-treated cells were centrifuged at 2,000 rpm for 5 min, then washed twice with sterile f/2 medium by centrifuging at 2,000 rpm for 5 min and removal of supernatant fluid. The cells were then transferred to conventional f/2 media for 8 days, with four or five rounds of continual transfer. Bacterial contamination was checked *via* traditional agar plate culturing.

### Co-culture Experiments

Bacteria were plated on fresh marine agar plates and grown from single colonies in marine broth by incubating for 72 h at 28°C in the dark with shaking at 200 rpm. Cells were then centrifuged at 8,000 rpm for 5 min and washed twice with sterile seawater, followed by diluting to a stock cell density of 10^1^−1 × 10^7^ cells/ml. Bacterial isolates and axenic *P. globosa* were then co-cultured using f/2 medium. The initial bacterial cell densities were 1 × 10^5^, 1 × 10^6^, and 1 × 10^7^ cells/ml, while *P*. *globosa* cell densities were adjusted to 1 × 10^4^ cells/ml to achieve starting bacterial: *P*. *globosa* ratios of 10:1, 100:1, and 1,000:1. Cultures with different starting ratios were designated as L (low cell density, 1 × 10^5^ cells/ml), M (medium cell density, 1 × 10^6^ cells/ml), and H (high cell density, 1 × 10^7^ cells/ml). In addition, *P*. *globosa* GX and ST strain cultures without bacterial co-culture were used as controls. All treatments and controls were conducted in triplicate. Cultures were maintained under the same conditions as stock cultures for 10 days (Liang et al., 2020). The abundances of solitary and colonial *P*. *globosa* cells were then counted using an Olympus inverted microscope (CKX53, Japan).

### DNA Extraction

A 400 ml sample of culture was used for DNA extractions, with three parallel replicates each. Samples were filtered with 0.22 μm Millipore filters to capture bacterial communities, followed by storage of filters at −20°C for subsequent DNA extraction. DNA extraction using the FastDNA Spin Kit for Soil (MP Biomedicals, Solon, OH, United States) according to the manufacturer’s instructions. DNA yield and purity were measured using a NanoDrop 2000 spectrophotometer (Thermo Scientific Inc., Waltham, MA, United States) and 1.0% agarose gel electrophoresis, respectively.

### High-Throughput Sequencing and Bioinformatics

The V3–V4 hypervariable regions of bacterial 16S rRNA gene were amplified using the universal primer with 338F/806R (Xu et al., 2016). PCRs comprised TransStart^®^ Fastpfu DNA Polymerase (TransGen Biotech, Beijing, China) and reactions were prepared according to the manufacturer’s instructions. PCR conditions included 95°C for 3 min followed by 27 cycles of 95°C for 30 s, 55°C for 30 s, and 72°C for 45 s, with a final extension at 72°C for 10 min. High-throughput sequencing of amplicons was conducted at Shanghai Majorbio Bio-pharm Co., Ltd. (Shanghai, China) on the Illumina MiSeq platform (Illumina, San Diego, CA, United States) with 250 bp paired-end sequencing.

Sequences were merged using FLASH (version 1.2.7) (Magoc and Salzberg, 2011) and the raw FASTQ files were quality filtered using fastp (version 0.20.0) (Chen et al., 2018). Quality filtered sequences were aligned with the SLIVA alignment (Quast et al., 2013), and sequences annotated as chloroplasts or mitochondria were removed, followed by clustering of sequences into operational taxonomic units (OTUs) at the 97% nucleotide sequence similarity threshold with UPARSE (version 7.1) (Edgar, 2013). Linear discriminant analysis Effect Size (LEfSe) analysis (Segata et al., 2011) was used to explore potential bacterial biomarkers associated with different *P*. *globosa* strains. The potential functions of bacterial populations were evaluated with FAPROTAX (version 1.2.4) (Louca et al., 2016).

### Statistical Analyses

The OTU sequence numbers were normalized to an equal number by 31065 for the later statistical analysis. The α-diversity indexes for richness estimators (ACE and Chao1), diversity (Shannon and Simpson), and Good’s coverage were calculated by using “vegan” R package (Jari Oksanen et al., 2019). The NMDS (non-metric multidimensional scaling) were by ANOSIM with 999 permutations for Bray–Curtis dissimilarities by using vegan and ggplot2 R packages (Wickham, 2017; Jari Oksanen et al., 2019). Distance-based redundancy analysis (db-RDA) followed the ANOVA with 999 permutations by using vegan R package (Jari Oksanen et al., 2019). Using the variable inflation factor (VIF) index with a maximum cut-off score of 10 checked multicollinearity among solitary cells abundance, colonial abundance, and colony diameter of *P*. *globosa*.

## Results

### Bacterial Community Diversity and Composition in the Guangxi and Shantou Strain Co-cultures

**FIGURE 1:**
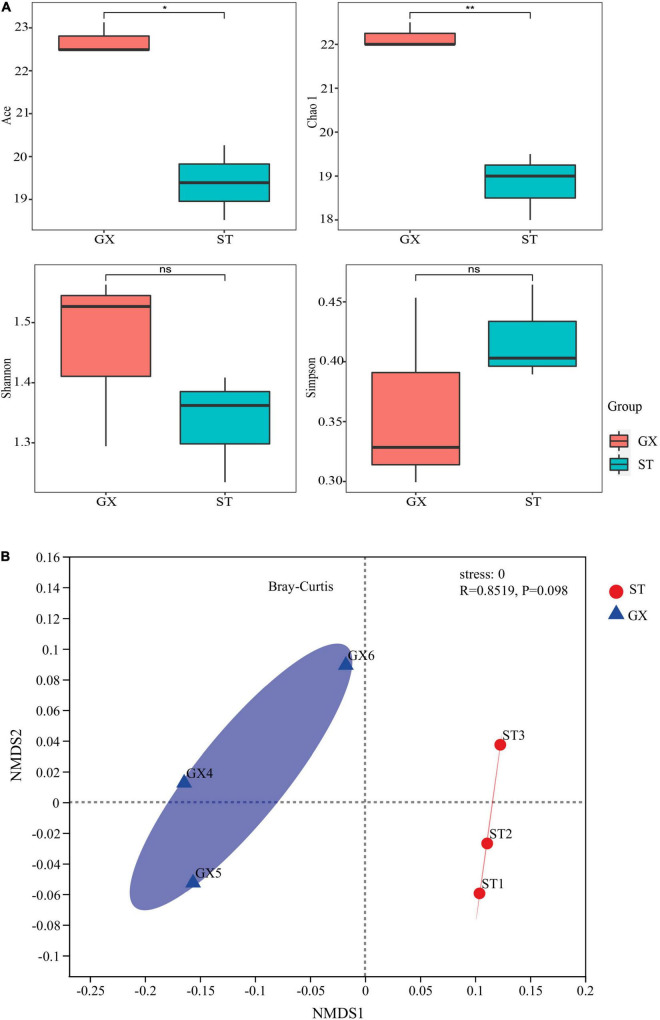
The diversity of bacterial communities associated with *Phaeocystis globosa* GX and ST strain cultures. **(A)** α-Diversity indices for the two co-culture communities. Statistical differences between pairs were assessed by *t*-tests. *^ns^*Not significant; *p < 0.05; **p < 0.01. **(B)** Non-parametric multidimensional scaling (NMDS) plots show variation in OTU compositions among the two co-cultures. GX, *P*. *globosa* GX strain co-culture; ST, *P*. *globosa* ST strain co-culture.

Rarefaction curves and Good’s coverage index indicated that the level of sequencing conducted was adequate to recover most sample diversity for both the GX and ST strain co-cultures (Supplementary Figure 1). The ACE and Chao 1 richness index values for the GX co-culture bacterial community were much higher than those of the ST strain, indicating that bacterial richness was significantly different in the GX and ST strain co-cultures (ACE, *p* < 0.05, Chao 1, *p* < 0.01). Significant differences were not observed for the Shannon and Simpson indices when comparing the GX and ST communities (Figure 1A), which is consistent with their bacterial OTU compositions (ANOSIM, *p* > 0.05 for Bray–Curtis metrics) through the NMDS analysis (Figure 1B).

**FIGURE 2:**
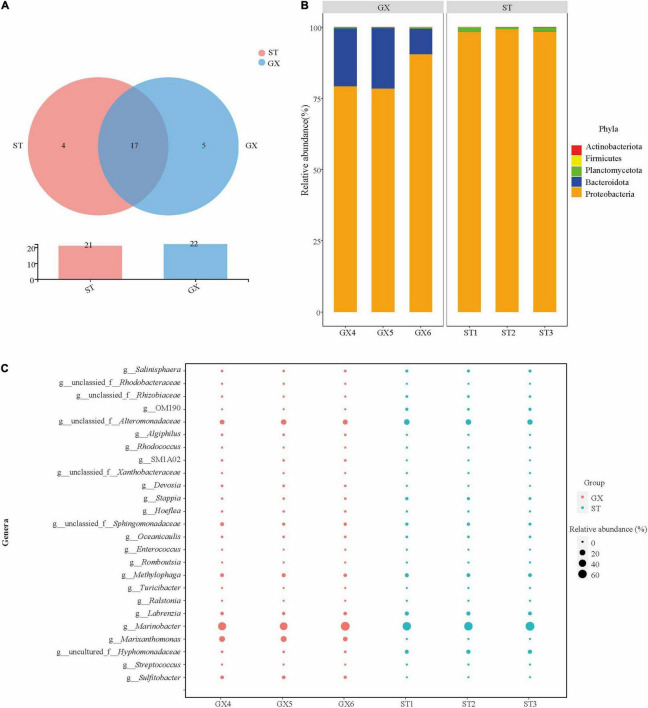
Composition of *Phaeocystis globosa* bacterial communities. **(A)** Shared and unique OTUs among co-cultures, visualized with Venn diagrams. **(B)** Co-culture bacterial community composition at the phylum level. **(C)** Co-culture bacterial community composition at the genus level. GX, *P*. *globosa* GX strain co-culture; ST, *P*. *globosa* ST strain co-culture.

A total of 26 OTUs were identified from the 6 co-culture (ST1, ST2, ST and GX4, GX5, GX6) communities, comprising 5 phyla, 8 classes, 15 orders, 21 families, and 25 genera. OTU2 was the dominant OTU among all samples (Supplementary Table 1). Community composition was considerably similar in both GX and ST co-cultures (Figure 2A). At the phylum level, Proteobacteria (82.7% average relative abundance) dominated the bacterial communities in both co-cultures, followed by Bacteroidota (16.9%), which were more abundant in the GX co-cultures. Proteobacteria (98.8%) and Planctomycetota (1.2%) were more abundant in the ST co-cultures, while Bacteroidota was absent. Proteobacteria was the most abundant phylum for both GX and ST co-cultures (Figure 2B). *Marinobacter*, *Marixanthomonas*, and unclassified *Alteromonadaceae* were the dominant genera in the GX co-culture (abundances >5%), while *Marinobacter*, unclassified *Alteromonadaceae*, uncultured *Hyphomonadaceae*, and *Labrenzia* were the dominant genera in the ST co-culture. Although the bacterial compositions differed between the GX and ST co-cultures, *Marinobacter* dominated both systems, with 55.0% and 61.9% abundances in the GX and ST co-cultures, respectively (Figure 2C).

### Bacterial Biomarkers in the Guangxi and Shantou Strain Co-cultures

**FIGURE 3:**
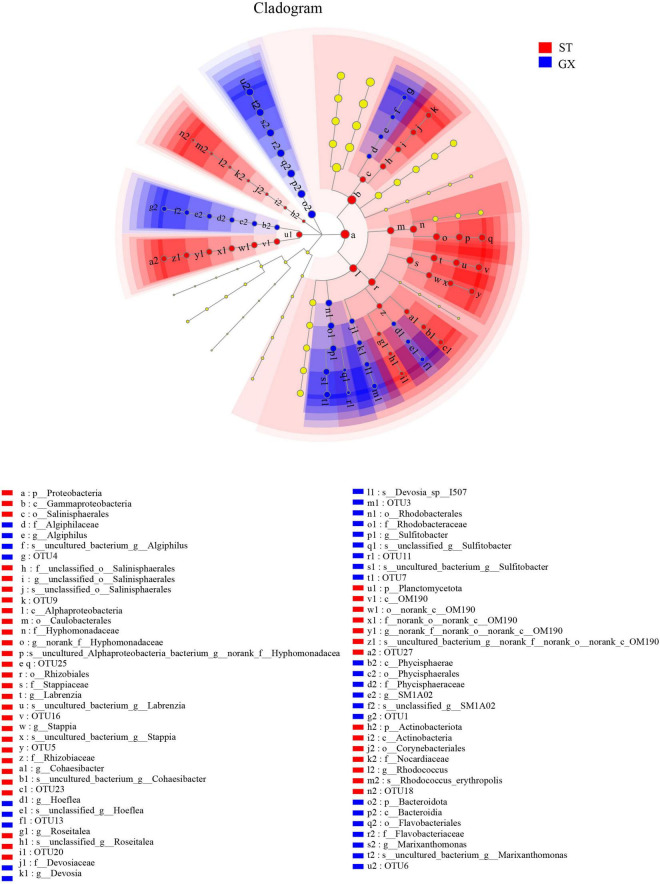
Cladogram showing the phylogenetic distributions of bacterial taxa associated with *Phaeocystis globosa* cultures. Circles indicate the taxonomic level ranging from the phylum to OTU levels. The diameter of each circle is proportional to the abundance of that group. GX, *P*. *globosa* GX strain co-culture; ST, *P*. *globosa* ST strain co-culture. p, phylum; o, order; f, family; g, genus; s, species.

**FIGURE 4:**
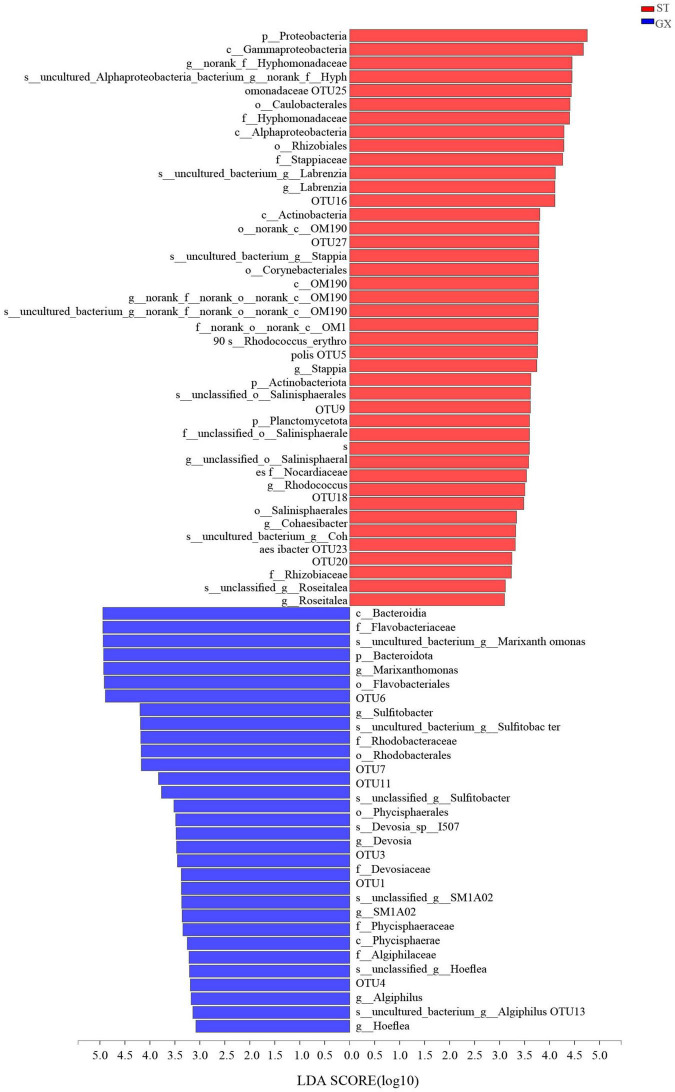
Indicator bacterial taxa with LDA scores >2. Different-colored regions of the bars in panel indicate taxa from different co-cultures. GX, *P*. *globosa* GX strain co-culture; ST, *P*. *globosa* ST strain co-culture. p, phylum; o, order; f, family; g, genus; s, species.

Cladograms were used to depict the distributions of taxonomic groups (Figure 3), with LDA scores >2 being used to identify biomarkers with LEfSe analysis (Figure 4). More biomarkers (LDA > 2) were identified for the *P*. *globosa* ST co-cultures compared to the GX co-cultures. Specifically, LEfSe analysis indicated that the *P*. *globosa* ST co-cultures included eight biomarkers including OTU27 (p__Planctomycetota, c__OM190), OTU20 (g__*Roseitalea*), OTU23 (g__*Cohaesibacter*), OTU5 (g__*Stappia*), and OTU16 (g__*Labrenzia*) affiliated with *Rhizobiales* (p_Proteobacteria, c_*Alphaproteobacteria*); OTU18 (g__*Rhodococcus*) affiliated with the *Corynebacteriales* (p__Actinobacteriota, c__*Actinobacteria*); OTU25 affiliated with the *Caulobacterales* (p__Proteobacteria, c__Alphaproteobacteria); and OTU9 (g__unclassified_o__*Salinisphaerales*) affiliated with the *Salinisphaerales* (p__Proteobacteria, c__Gammaproteobacteria).

The *P*. *globosa* GX co-culture harbored seven biomarkers including OTU3 (g_*Devosia*) and OTU13 (g_*Hoeflea*) affiliated with the *Rhizobiales* (p_Proteobacteria, c_*Alphaproteobacteria*); OTU11 and OTU7 (g_*Sulfitobacter*) affiliated with the *Rhodobacterales* (p_Proteobacteria, c_*Alphaproteobacteria*); along with OTU6 (g__*Marixanthomonas*), OTU1 (g__SM1A02), and OTU4 (g__*Algiphilus*). Greater numbers of biomarkers affiliated with *Rhizobiales* were enriched in the *P*. *globosa* ST co-cultures compared to the GX co-culture, while one biomarker was affiliated with the Planctomycetota in *P*. *globosa* ST co-culture that was not observed in the GX co-cultures.

### Potential Metabolic Functions of Bacterioplankton in the Guangxi and Shantou Strain Co-cultures

**FIGURE 5:**
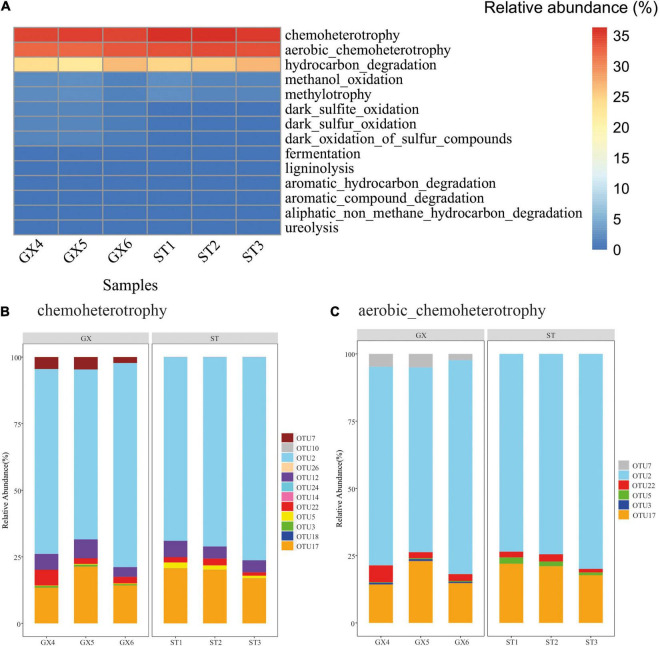
Predicted functional groups among the bacterial communities associated with *Phaeocystis globosa* cultures. **(A)** Relative abundances of functional classifications. **(B)** OTUs associated with predicted chemoheterotrophic functions. **(C)** OTUs associated with predicted aerobic chemoheterotrophic functions.

FAPROTAX was used to predict the potential metabolic functions of bacterioplankton populations based on 16S rRNA gene identities. Twelve functional groups were predicted from the 26 OTUs. Chemoheterotrophic (chemoheterotrophy and aerobic chemoheterotrophy) microbial populations were predicted as the most dominant groups, accounting for 67.7 and 70.3% of the *P. globosa* GX and ST co-cultures, respectively (Figure 5A). Hydrocarbon degradation-associated functional groups accounted for 24.3 and 26.0% of the *P. globosa* GX and ST co-cultures, respectively. OTU2 (*Marinobacter*) was present in all samples and was predicted to be involved in chemoheterotrophy, aerobic chemoheterotrophy, and hydrocarbon degradation (Figures 5B,C and Supplementary Table 2). Overall, functional predictions suggested that bacterial communities of *P. globosa* GX and ST co-cultures maintained similar metabolic functions, with the dominant functional profiles arising from *Marinobacter*.

### Co-culture Experiments of Bacterial and *Phaeocystis globosa* Strains

To identify strain-specific interactions between *Phaeocystis* and associated bacteria, ten and eight cultivable bacterial strains were isolated from the GX and ST co-cultures, respectively. The strains were identified as *Alteromonas*, *Hoeflea*, *Labrenzia*, *Sulfitobacter*, *Oceanicaulis*, and *Marinobacter* genera based on >97% similarity in 16S rRNA gene sequences.

*Marinobacter* GS7 cells are Gram-negative, rod-shaped, not flagellated, 1.5–2.5 μm in length, and 0.3–0.5 μm wide (Supplementary Figure 2). Phylogenetic analysis based on 16S rRNA gene sequences indicated that GS7 was closely related to *Marinobacter shengliensis* SL013A34A2 (98.6% 16S rRNA gene identity) (Supplementary Figure 3). GS7 formed a distinct phylogenetic cluster (16S rRNA genes exhibited 99.7% nucleotide similarity) with OTU2, which was the dominant taxa in GX and ST strain co-cultures (Supplementary Figure 4 and Supplementary Table 1).

**FIGURE 6:**
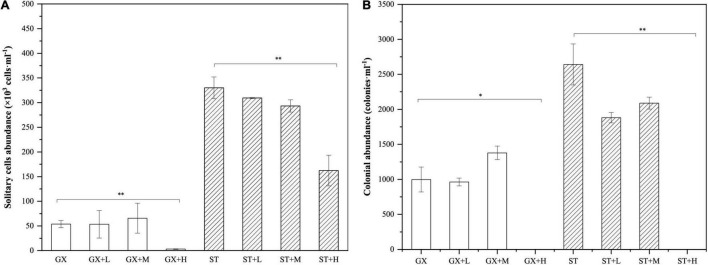
Growth characteristics of *Phaeocystis globosa-Marinobacter* sp. GS7 co-cultures. **(A)** The abundances of solitary *P. globosa* cells. **(B)** The abundances of *P. globosa* colonies. Each bar represents the mean of three biological replicates and error bars indicate standard deviations of the means. Statistical differences between treatments and controls were tested with *t*-tests. *p < 0.05; **p < 0.01. Non-significant comparisons are not marked. GX, *P*. *globosa* GX strain co-culture; ST, *P*. *globosa* ST strain co-culture. L, M, and H indicate the final bacterial cell densities of 1 × 10^5^, 1 × 10^6^, and 1 × 10^7^ cells/ml, respectively.

We first determined that the growth rate of *P. globosa* was not influenced by the short-term removal of bacterial populations. Further, the growth rate was also unaffected when co-cultured with *Marinobacter* sp. GS7 at cell densities of 1 × 10^1^ to 1 × 10^4^ cells/ml. However, high cell densities of *Marinobacter* sp. GS7 significantly decreased (*p* < 0.01) the solitary cell abundances of *P. globosa* ST and GX strains (Figure 6A). In contrast, low and medium cell densities of *Marinobacter* sp. GS7 did not impact (*p* > 0.05) solitary cell abundances of the *P*. *globosa* ST and GX strains (Figure 6A). Addition of low and medium cell densities of *Marinobacter* sp. GS7 also did not impact (*p* > 0.05) *P*. *globosa* colony numbers for either strain. Rather, *P*. *globosa* GX and ST strains failed to form colonies when they were exposed to high cell densities of *Marinobacter* sp. GS7 (Figure 6B).

## Discussion

### *Phaeocystis globosa* Effects on Bacterial Assembly

Bacterial communities associated with field *P*. *globosa* blooms were studied for decades, while bacterial community assemblage and function associated with long-term *P*. *globosa* laboratory culture remain relatively understudied (Li et al., 2020; Rosenberg et al., 2021). Marine environmental conditions change dynamically across multiple temporal and spatial scales, thereby impacting bacterial communities (Rosenberg et al., 2021). These selective forces can drive divergent bacterial community succession in distinct natural marine environments (Jasti et al., 2005). In contrast to marine environments, laboratory culture conditions can be altered to modulate single variables including light, temperature, and the concentrations of dissolved inorganic and organic nutrients. In this study, only 26 bacterial OTUs were obtained from laboratory co-cultures, representing a simplified microbial community compared to communities in natural marine ecosystems. Many studies have shown that bacterial communities associated with *P*. *globosa* blooms are dominated by Proteobacteria and Bacteroidota (Brussaard et al., 2005; Buchan et al., 2014; Delmont et al., 2014; Wemheuer et al., 2015; Wohlbrand et al., 2017; Li et al., 2020). Moreover, Guangxi and Shantou are two distinct coastal systems, which are shaped by tides, weather, and local anthropogenic influences (Wang et al., 2013). For instance, the sea surface temperature and light intensity of Guangxi are usually higher than that of Shantou (Zhao et al., 2018), thus, primary production in Guangxi is relatively higher than Shantou over the whole year (Ma et al., 2021). These distinct local environments select for adapted *P. globosa* strains, which further select associated microbes. In laboratory cultures, this close phytoplankton-heterotroph association was maintained and enhanced after many generations. In this study, Proteobacteria were also the dominant bacteria phylum among all *P*. *globosa* long-term laboratory cultures, while Bacteroidota were not observed in the *P*. *globosa* ST strain co-culture. The abundances of Bacteroidota (OTU6) were positively correlated with colony abundances but were negatively correlated with solitary cell abundances by db-RDA analysis, suggesting Bacteroidota may be associated with *P*. *globosa* colonies. ST co-cultures are dominated by solitary cells in life history (Liang et al., 2020), which may not favor the attachment and growth of Bacteroidota, thus gradually lost during the *P*. *globosa* cultivation.

Since laboratory culture conditions of both strains were identical, the difference in bacterial community assemblage likely arose from selective forces imposed by differences in the two *P*. *globosa* strains. In addition, the most dominant bacterial genus, *Marinobacter*, as identified in this study, was not the most dominant one in previous studies which used *P*. *globosa* strains differ from the current study (Xu et al., 2021a,b; Zhu et al., 2021). These results suggest that different *P*. *globosa* strains might recruit different microbes, and stable association could be formed during the long-time laboratory co-culture (Kogure et al., 1982; Vaqué et al., 1990; Sapp et al., 2007).

**FIGURE 7:**
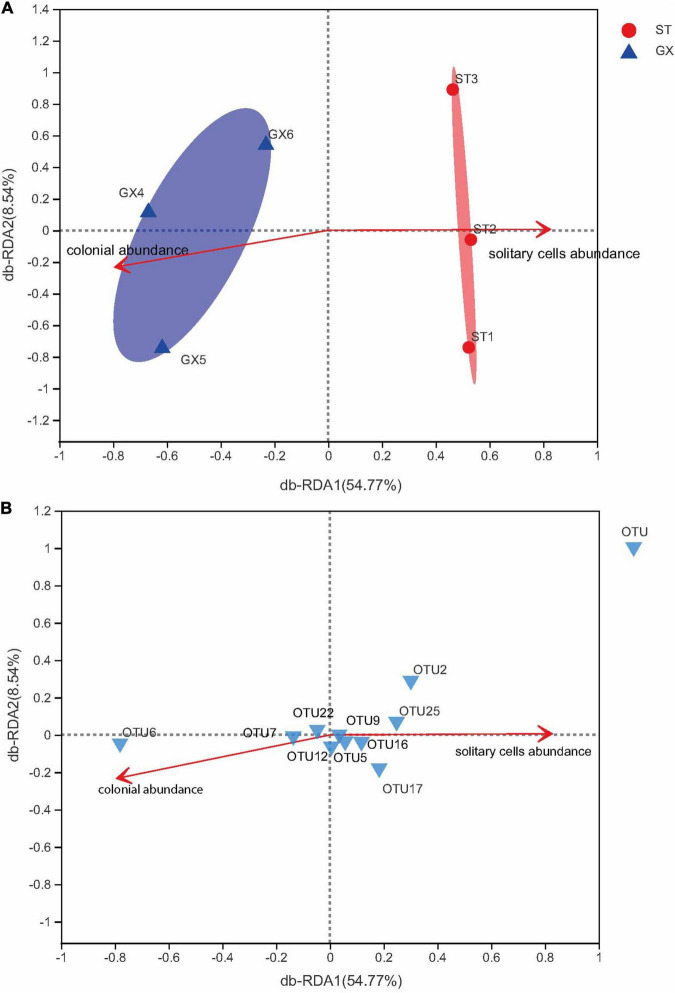
Distance-based redundancy analysis (db-RDA) ordination plots of bacterial community variation at the OTU level. **(A)** The relationships of *Phaeocystis globosa* growth indices and community composition. The relationships of *P. globosa* growth indices and the 10 most abundant OTUs **(B)**. GX, *P*. *globosa* GX strain co-culture; ST, *P*. *globosa* ST strain co-culture.

In addition, db-RDA modeling was used to assess the associations of *P*. *globosa* solitary cell abundances and colony abundances as meaningful explanatory variables of bacterial community variation (Figure 7A). Bacterial communities of *P*. *globosa* GX cultures were associated with *P. globosa* colony abundances. In contrast, bacterial communities of *P*. *globosa* ST cultures differed from GX co-cultures and were associated with *P*. *globosa* solitary cell abundances. Thus, bacterial communities are impacted by *P*. *globosa* growth dynamics and strain-specific characteristics. These results are consistent with previous studies indicating that bacterial community composition is selected by characteristics of microalgal growth, but also by characteristics of different microalgal strains (Kogure et al., 1982; Vaqué et al., 1990; Sapp et al., 2007).

In the present study, the potential functions of bacteria associated with *P*. *globosa* were evaluated using the FAPROTAX software. Chemoheterotrophy was the dominant predicted metabolic lifestyle among *P. globosa* co-culture bacteria, mostly contributed by *Marinobacter*. Likewise, db-RDA (Figure 7B) indicated that bacterial community composition (considering the 10 most abundant OTUs) varied with *P*. *globosa* growth. Specifically, the abundances of *Marinobacter* (OTU2) were positively correlated with solitary cell abundances but were negatively correlated with colony abundances, suggesting *Marinobacter* is associated with *P*. *globosa* growth.

Linear discriminant analysis Effect Size analysis indicated that the *P*. *globosa* GX and ST co-cultures were associated with different biomarkers. Interestingly, both *P*. *globosa* strains were associated with diverse N_2_-fixing bacteria, with five N_2_-fixing OTUs identified as biomarkers in the ST cultures, and two N_2_-fixing OTUs in the GX strain. These bacteria included Planctomycetota that encode *nifD* and *nifH* genes (Delmont et al., 2018) and *Alphaproteobacteria* (order Rhizobiales) that include N_2_-fixing rhizobia (Remigi et al., 2016; Zhang et al., 2021). Besides the N_2_-fixing bacterial biomarkers (*Rhizobiales*), *P*. *globosa* GX co-culture also harbored an OTU affiliated with *Sulfitobacter* which was not absent in the *P*. *globosa* ST co-culture. Previous studies Hypothesized that N_2_-fixing bacteria can fix nitrogen, which might release the nitrogen limitation of algae (Ferro et al., 2019; Tait et al., 2019). In addition, studies also showed that *Sulfitobacter* could enhance the growth of *Pseudo-Nitzschia multiseries via* secretion of the hormone indole-3-acetic acid (Amin et al., 2015; Seymour et al., 2017). Thus, long-term cultivation of *P*. *globosa* leads to the selection of bacteria that could be beneficial for the dominant microalgae.

### *Marinobacter* Negatively Affects *Phaeocystis globosa* Colony Development

A previous study also indicated that *Marinobacter* might be a potential bacterial bioindicator of *P*. *globosa* blooms (Li et al., 2020). *Marinobacter* exhibits high metabolic diversity, that can use a variety of carbon sources, and participate in important biogeochemical cycling processes (Bonis and Gralnick, 2015; Wang et al., 2015; Wemheuer et al., 2015). Besides, *Marinobacter* also plays a major role as a dominant Fe (II)-oxidizer in different environments (Bonis and Gralnick, 2015). *Marinobacter* can be found in single algal laboratory cultures, but also coexist with microalgae (Alavi et al., 2001; Hold et al., 2001b; Lupette et al., 2016).

An association of *P*. *globosa* solitary cell numbers and colony abundances was observed with *Marinobacter* sp. GS7 when grown in co-culture. Interestingly, co-culture with low and medium cell densities of *Marinobacter* did not lead to significant changes in *P. globosa* solitary cell and colony abundances, while high cell densities of *Marinobacter* sp. GS7 significantly impaired the growth and colony formation of *P. globosa*. The inhibition effect on *P. globosa* growth might be related to the physiological change of *Marinobacter* cells at high cell densities compared to low cell densities, such as chemotaxis, cell motility, and attachment (Sonnenschein Eva et al., 2012), or extracellular electron transfer (Eddie et al., 2021), or discharging of chemical mediators such as antibacterial and algicidal compounds (Imai et al., 1993; Mayali and Azam, 2004; Meyer et al., 2017; Cirri and Pohnert, 2019). Lifestyle change of *Alteromonas*, another opportunitroph frequently associated with marine phytoplankton, has been observed when co-culturing with *Trichodesmium* (Hou et al., 2018). *Alteromonas* cell motility and cellular activities were tightly regulated and coupled with the physiology of phytoplankton. Here the density-dependent inhibitory effect of *Marinobacter* on *P. globosa* growth might indicate a transition of their relationship from mutualism/commensalism to competition. When *Marinobacter* cell density was low, *P. globosa* exudates facilitated the heterotrophic growth of *Marinobacter* as important carbon sources (Fouilland et al., 2014). In return, *Marinobacter* may produce growth factors or secrete siderophores to promote the growth of associated phytoplankton (Töpel et al., 2019). At high cell densities, *Marinobacter* cells may compete with *P. globosa* for resources or compete with each other for attachment to phytoplankton, thus preventing *P. globosa* growth and colony formation. Previous studies reported that many heterotrophic bacteria could secrete chemicals inhibiting phytoplankton growth (Gallacher et al., 1997; Hold et al., 2001a; Kim et al., 2008; Yang et al., 2014). Some of the algicidal compounds are concentration dependent (Paul and Pohnert, 2011; Tan et al., 2016), which are likely to release at high bacterial cell densities (Mayali and Doucette, 2002; Roth et al., 2008) for competing nutrients (Meyer et al., 2017). Thus, it is possible that at high cell densities, algicidal compounds or other toxic substances produced by *Marinobacter* inhibited the growth and colony formation of *P. globosa*. Although we could not determine the exact chemicals in this study, it represents an interesting avenue for future research.

## Conclusion

In this study, bacterial communities associated with two *P. globosa* strains and their influence on colony formation were evaluated. The *P. globosa* GX strain cultures showed higher bacterial community richness than the ST strain cultures. Overall community compositions and biomarker bacteria were also different between the two *P. globosa* strains, suggesting a strain-specific epibiont association. Co-culture experiments with *P. globosa* and *Marinobacter* sp. GS7 revealed that *P*. *globosa* formed colonies in the presence of low and medium cell densities of *Marinobacter*, but high cell densities of *Marinobacter* severely inhibited colony formation. In summary, these results support the hypothesis that bacteria communities associated with *P. globosa* strains are strain-specifically selected, and associated bacterioplankton could play key roles in *P. globosa* colony formation processes.

## Data Availability Statement

The datasets presented in this study can be found in online repositories. The names of the repository/repositories and accession number(s) can be found in the article/Supplementary Material.

## Author Contributions

SX, XW, Z-HW, and YW designed the experiment and prepared the manuscript. JL and FZ prepared the sampling and co-culture experiments. SX, KG, and SC conducted the sequencing and bioinformatics analyses. All authors contributed to the article and approved the submitted version.

## Conflict of Interest

The authors declare that the research was conducted in the absence of any commercial or financial relationships that could be construed as a potential conflict of interest.

## Publisher’s Note

All claims expressed in this article are solely those of the authors and do not necessarily represent those of their affiliated organizations, or those of the publisher, the editors and the reviewers. Any product that may be evaluated in this article, or claim that may be made by its manufacturer, is not guaranteed or endorsed by the publisher.

## Footnotes

1
https://www.ezbiocloud.net/
