# Supplementary material for: Bacteria Associated With Phaeocystis globosa and Their Influence on Colony Formation
Source: Front Microbiol. 2022 Feb 17;13:826602. doi: 10.3389/fmicb.2022.826602 (PMC8891983; doi:10.3389/fmicb.2022.826602)
Supplement: Supplementary file 1 [file Data_Sheet_1.PDF]

Supplementary Material

Bacteria associated with *Phaeocystis globosa* and their influence on colony formation

Shuaishuai Xu[1†], Xiaodong Wang[1†], Jie Liu[1], Fengli Zhou[1], Kangli Guo[2], Songze Chen[2], Zhao-hui Wang[1]*, Yan Wang[1]*

[1] College of Life Science and Technology, Jinan University, Guangzhou 510632, China

[2] Department of Ocean Science and Engineering, Southern University of Science and Technology, Shenzhen 518055, China

***Correspondence:**
Zhao-Hui Wang. E-mail address: twzh@jnu.edu.cn
Yan Wang. E-mail address: wangyan@jnu.edu.cn

[†] These authors have contributed equally to this work.

Supplementary Figure 1. Rarefaction curves for all samples. GX, *P. globosa* GX strain co-culture; ST, *P. globosa* ST strain co-culture.

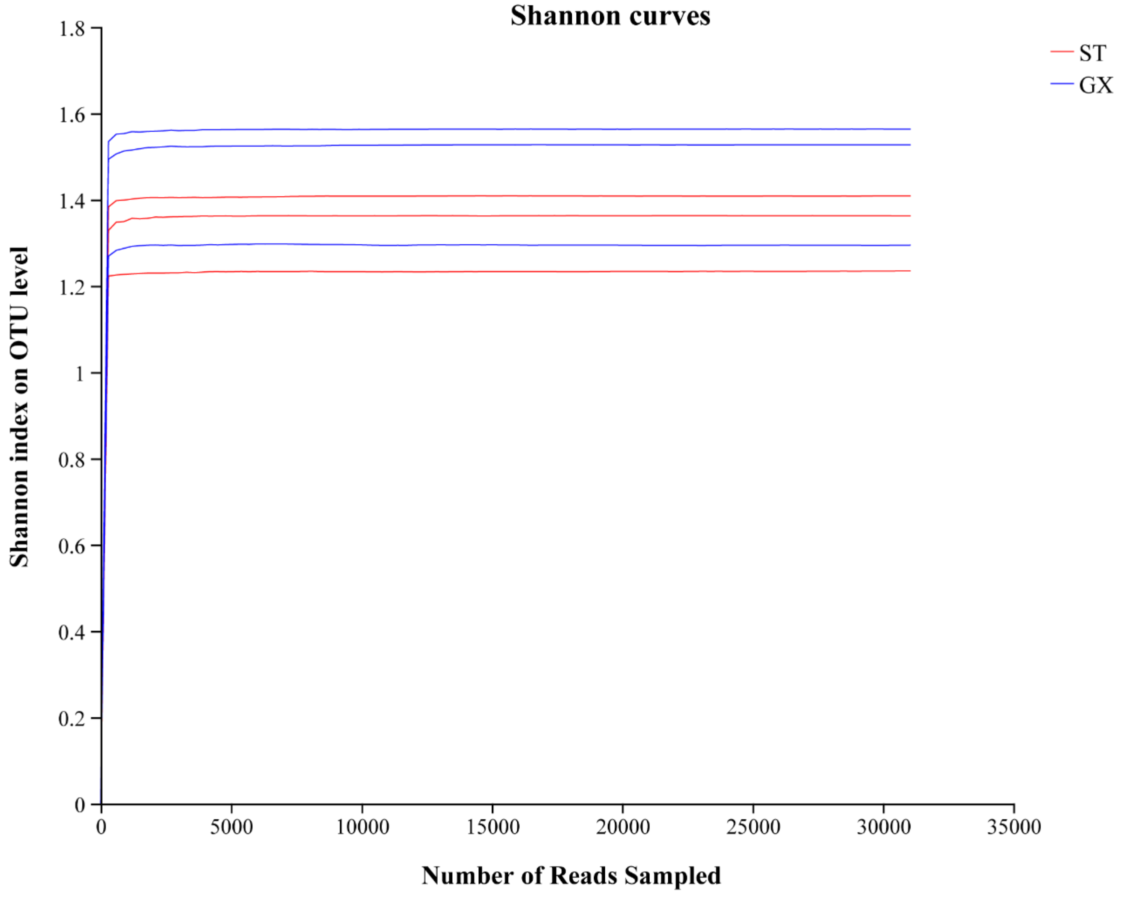

Supplementary Figure 2. *Marinobacter* sp. GS7 cellular morphologies. Cells were cultivated on marine agar 2216 (MA; BD Difco) at 28°C for 72 h, fixed with glutaraldehyde, and photographed with a scanning electron microscope (Zeiss ULTRA^{TM} 55, Carl Zeiss Inc., Oberkochen, Germany). (a) Bar: 1 μm. (b) Bar: 200 nm.

(a)
(b)

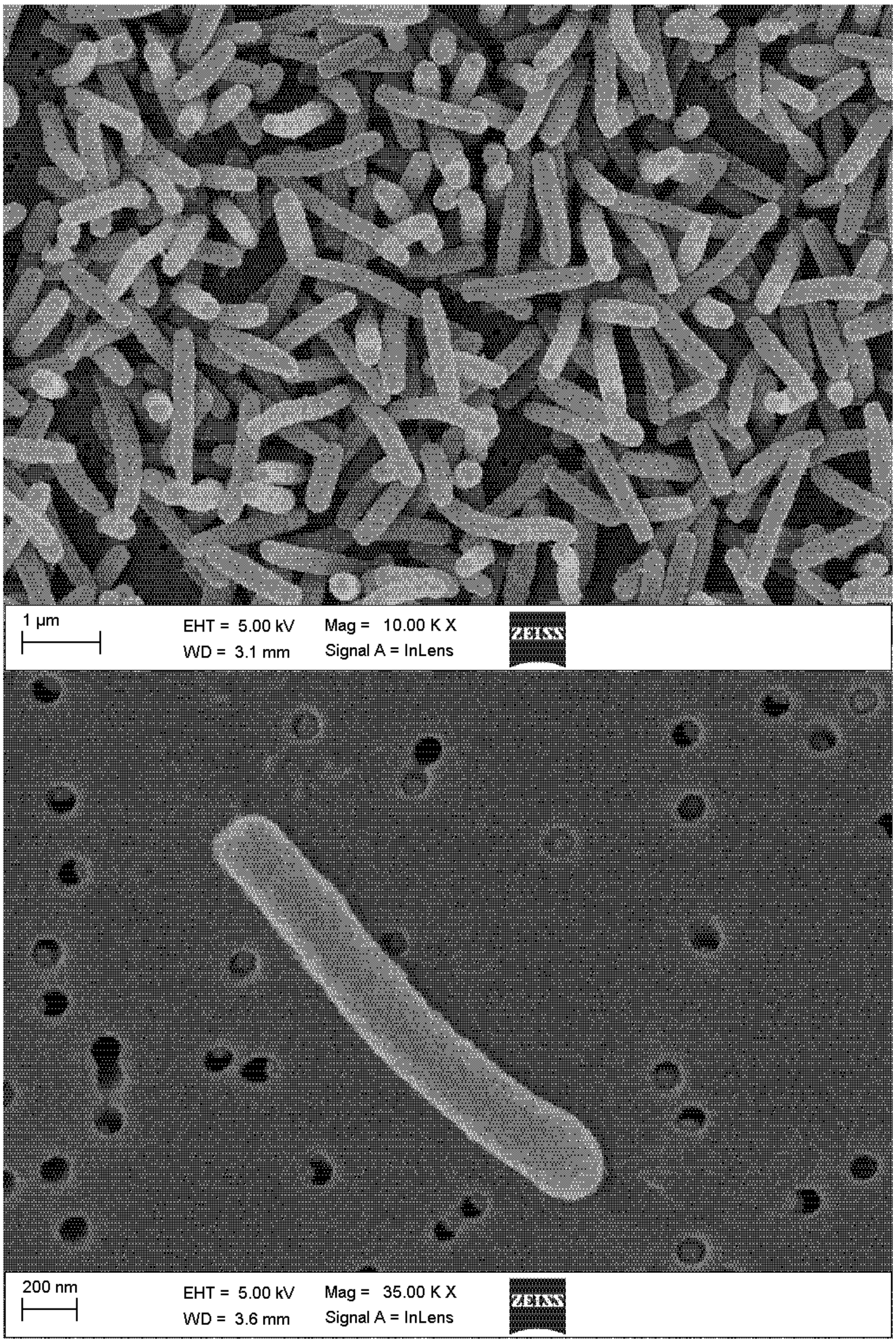

Supplementary Figure 3. Maximum Likelihood 16S rRNA gene phylogeny showing *Marinobacter* sp. GS7 relationships to other congeners. Bootstrap values are based on 1,000 replicates and are shown at branch nodes. Bootstrap values below 50% are not shown. Bar = 0.01 substitutions per nucleotide position.

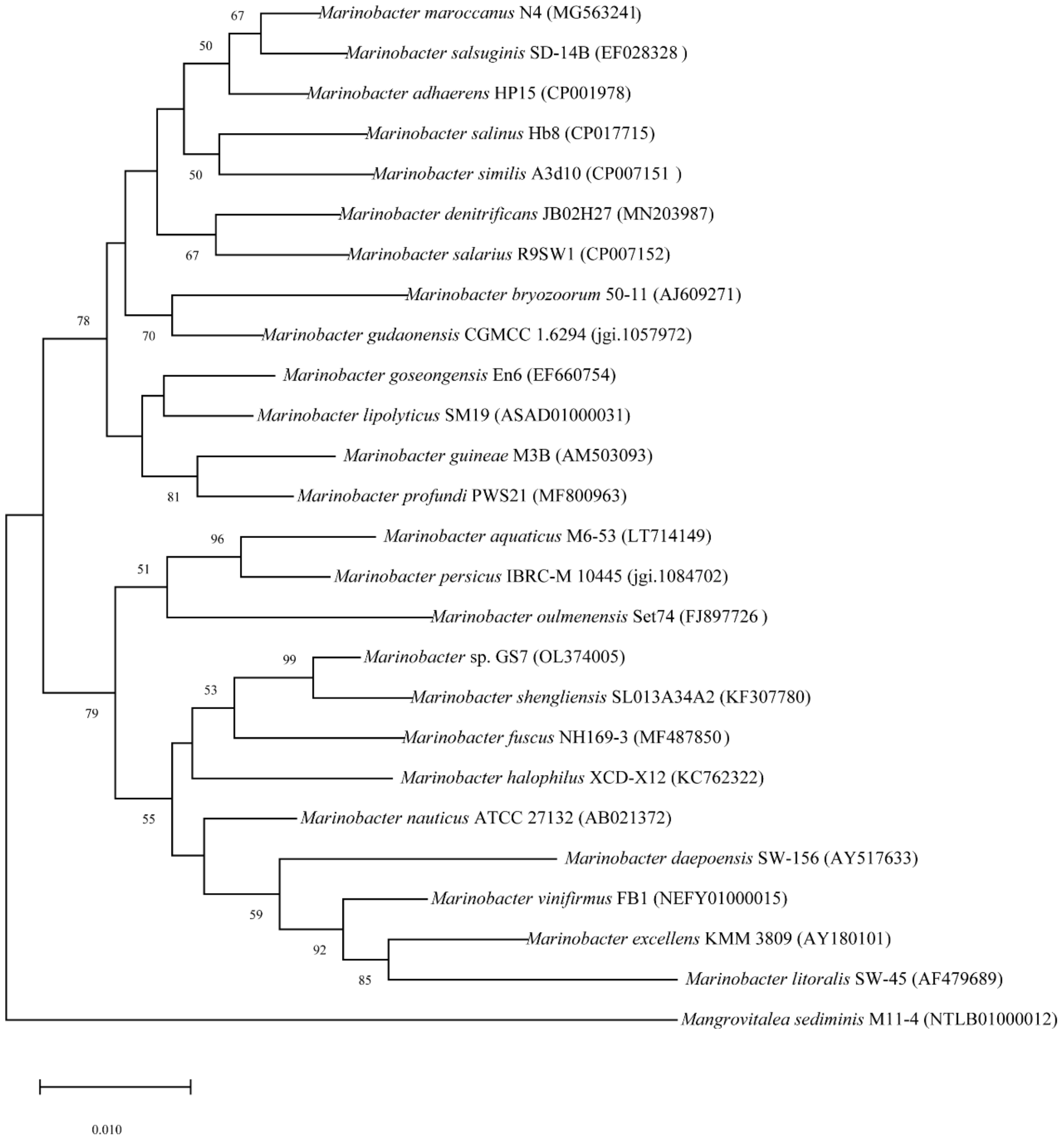

Supplementary Figure 4. Maximum Likelihood 16S rRNA gene phylogeny showing relationships among OTUs recovered in this study. Bootstrap values are based on 1,000 replicates and are shown at branch nodes. Bootstrap values below 50% are not shown. Bar = 0.05 substitutions per nucleotide position.

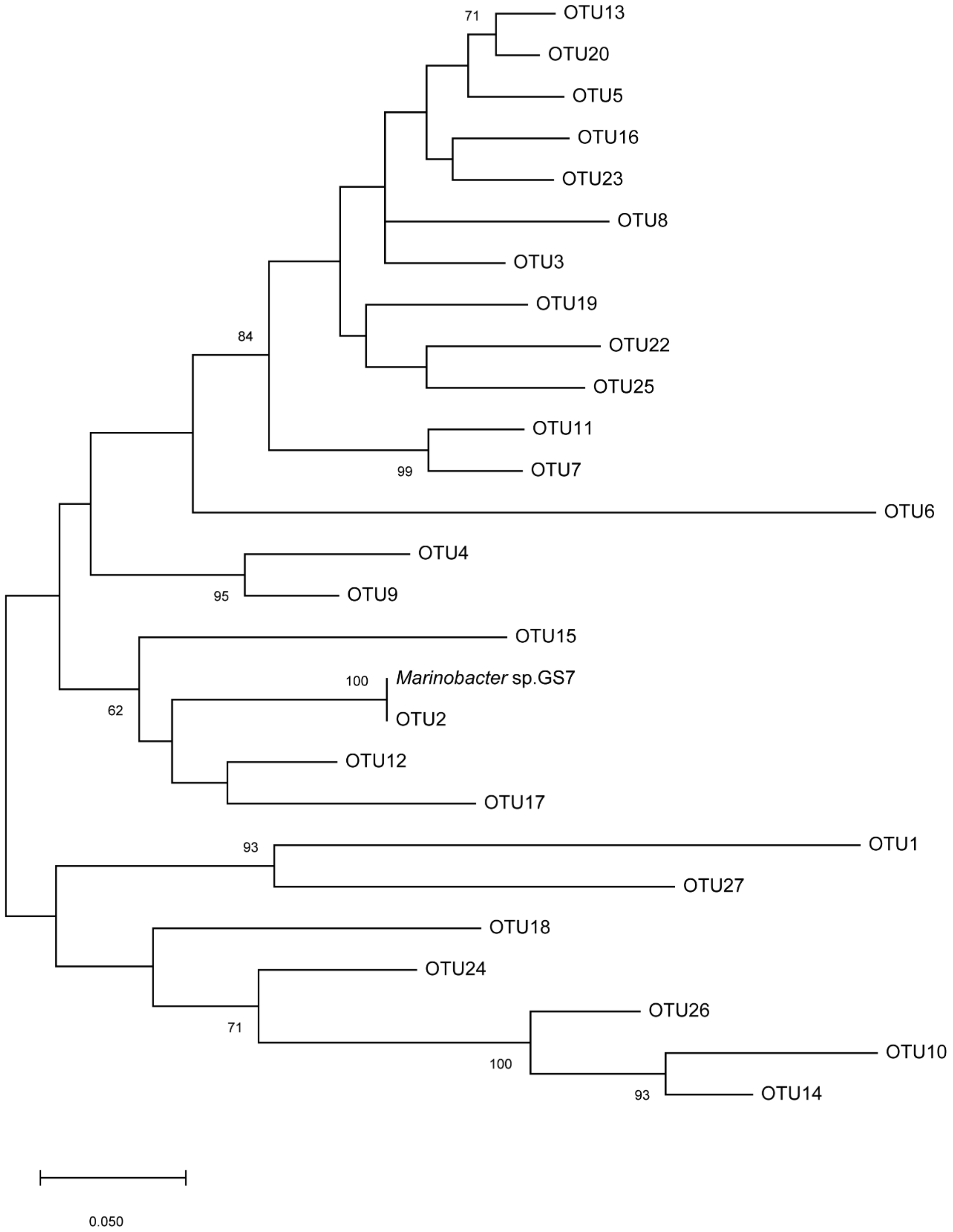

Supplementary Table 1. Taxonomic composition of *Phaeocystis globosa*-associated bacterial communities.

#OTU ID	GX4	GX5	GX6	ST1	ST2	ST3	Taxonomy
OTU9	189	180	247	393	467	344	d__Bacteria; p__Proteobacteria; c__Gammaproteobacteria; o__Salinisphaerales; f__Salinisphaeraceae; g__Salinisphaera; s__uncultured_bacterium
OTU11	11	39	18	0	0	0	d__Bacteria; p__Proteobacteria; c__Alphaproteobacteria; o__Rhodobacterales; f__Rhodobacteraceae
OTU23	8	1	8	164	182	89	d__Bacteria; p__Proteobacteria; c__Alphaproteobacteria; o__Rhizobiales; f__Rhizobiaceae
OTU27	0	0	0	563	249	431	d__Bacteria; p__Planctomycetota; c__OM190; o__OM190; f__OM190; g__OM190; s__uncultured_bacterium
OTU20	8	9	13	42	28	28	d__Bacteria; p__Proteobacteria; c__Alphaproteobacteria; o__Rhizobiales; f__Rhizobiaceae
OTU17	5720	10493	7411	6353	7358	4552	d__Bacteria; p__Proteobacteria; c__Gammaproteobacteria; o__Alteromonadales; f__Alteromonadaceae
OTU4	132	208	148	20	14	7	d__Bacteria; p__Proteobacteria; c__Gammaproteobacteria; o__Salinisphaerales; f__Algiphilaceae; g__Algiphilus; s__uncultured_bacterium
OTU18	7	4	1	5	5	3	d__Bacteria; p__Actinobacteriota; c__Actinobacteria; o__Corynebacteriales; f__Nocardiaceae; g__Rhodococcus
OTU1	187	198	259	0	0	0	d__Bacteria; p__Planctomycetota; c__Phycisphaerae; o__Phycisphaerales; f__Phycisphaeraceae; g__SM1A02; s__uncultured_bacterium
OTU8	3	0	2	0	5	1	d__Bacteria; p__Proteobacteria; c__Alphaproteobacteria; o__Rhizobiales; f__Xanthobacteraceae
OTU3	257	335	228	0	0	0	d__Bacteria; p__Proteobacteria; c__Alphaproteobacteria; o__Rhizobiales; f__Devosiaceae; g__Devosia
OTU5	70	137	147	680	600	255	d__Bacteria; p__Proteobacteria; c__Alphaproteobacteria; o__Rhizobiales; f__Stappiaceae; g__Stappia
OTU13	103	121	116	20	18	7	d__Bacteria; p__Proteobacteria; c__Alphaproteobacteria; o__Rhizobiales; f__Rhizobiaceae; g__Hoeflea
OTU22	2516	1043	1271	615	942	345	d__Bacteria; p__Proteobacteria; c__Alphaproteobacteria; o__Sphingomonadales; f__Sphingomonadaceae
OTU19	200	298	337	116	197	119	d__Bacteria; p__Proteobacteria; c__Alphaproteobacteria; o__Caulobacterales; f__Hyphomonadaceae; g__Oceanicaulis
OTU14	0	0	1	1	0	0	d__Bacteria; p__Firmicutes; c__Bacilli; o__Lactobacillales; f__Enterococcaceae; g__Enterococcus; s__Enterococcus_cecorum
OTU24	0	0	0	1	1	7	d__Bacteria; p__Firmicutes; c__Clostridia; o__Peptostreptococcales-Tissierellales; f__Peptostreptococcaceae; g__Romboutsia
OTU12	2545	3497	1915	1890	1665	1208	d__Bacteria; p__Proteobacteria; c__Gammaproteobacteria; o__Nitrosococcales; f__Methylophagaceae; g__Methylophaga; s__Methylophaga_sp.
OTU26	0	0	0	0	2	1	d__Bacteria; p__Firmicutes; c__Bacilli; o__Erysipelotrichales; f__Erysipelotrichaceae; g__Turicibacter
OTU15	11	9	11	12	7	2	d__Bacteria; p__Proteobacteria; c__Gammaproteobacteria; o__Burkholderiales; f__Burkholderiaceae; g__Ralstonia
OTU16	1679	1511	1954	2071	2449	1280	d__Bacteria; p__Proteobacteria; c__Alphaproteobacteria; o__Rhizobiales; f__Stappiaceae; g__Labrenzia
OTU2	29551	31345	39735	21212	25989	20500	d__Bacteria; p__Proteobacteria; c__Gammaproteobacteria; o__Alteromonadales; f__Marinobacteraceae; g__Marinobacter; s__Marinobacter_alkaliphilus
OTU6	11556	13984	5547	0	0	0	d__Bacteria; p__Bacteroidota; c__Bacteroidia; o__Flavobacteriales; f__Flavobacteriaceae; g__Marixanthomonas; s__uncultured_bacterium
OTU25	171	246	270	1685	2801	1886	d__Bacteria; p__Proteobacteria; c__Alphaproteobacteria; o__Caulobacterales; f__Hyphomonadaceae; g__uncultured
OTU10	4	6	4	4	2	0	d__Bacteria; p__Firmicutes; c__Bacilli; o__Lactobacillales; f__Streptococcaceae; g__Streptococcus; s__Streptococcus_hyointestinalis
OTU7	1906	2276	1154	0	0	0	d__Bacteria; p__Proteobacteria; c__Alphaproteobacteria; o__Rhodobacterales; f__Rhodobacteraceae; g__Sulfitobacter

GX, *P. globosa* GX strain co-culture; ST, *P. globosa* ST strain co-culture.

Supplementary Table 2. Predicted functions of OTUs based on FAPROTAX database classifications and the assignment to chemoheterotrophy, aerobic chemoheterotrophy, and hydrocarbon degradation functional groups.

# chemoheterotrophy						
#OTU ID	GX4	GX5	GX6	ST1	ST2	ST3
OTU17	5720	10493	7411	6353	7358	4552
OTU18	7	4	1	5	5	3
OTU3	257	335	228	0	0	0
OTU5	70	137	147	680	600	255
OTU22	2516	1043	1271	615	942	345
OTU14	0	0	1	1	0	0
OTU24	0	0	0	1	1	7
OTU12	2545	3497	1915	1890	1665	1208
OTU26	0	0	0	0	2	1
OTU2	29551	31345	39735	21212	25989	20500
OTU10	4	6	4	4	2	0
OTU7	1906	2276	1154	0	0	0
# aerobic_chemoheterotrophy						
#OTU ID	GX4	GX5	GX6	ST1	ST2	ST3
OTU17	5720	10493	7411	6353	7358	4552
OTU3	257	335	228	0	0	0
OTU5	70	137	147	680	600	255
OTU22	2516	1043	1271	615	942	345
OTU2	29551	31345	39735	21212	25989	20500
OTU7	1906	2276	1154	0	0	0
# hydrocarbon_degradation						
#OTU ID	GX4	GX5	GX6	ST1	ST2	ST3
OTU2	29551	31345	39735	21212	25989	20500
OTU18	7	4	1	5	5	3

GX, *P. globosa* GX strain co-culture; ST, *P. globosa* ST strain co-culture.
